# Women with Abdominal Aortic Aneurysms Have a Different Pattern of Genetic Variability, Compared to Men

**DOI:** 10.3390/biomedicines14051172

**Published:** 2026-05-21

**Authors:** Jonas Wallinder, Anders Wanhainen, Helena Åkerud, Dick Wågsäter, Martin Björck

**Affiliations:** 1 Department of Surgical Sciences, Section of Vascular Surgery, Uppsala University, 751 85 Uppsala, Sweden; anders.wanhainen@uu.se (A.W.); martin.bjorck@uu.se (M.B.); 2 Department of Surgery, Sundsvall District Hospital, 856 43 Sundsvall, Sweden; 3 Department of Immunology, Genetics and Pathology, Uppsala University, 751 85 Uppsala, Sweden; 4 Department of Medical Cell Biology, Uppsala University, 751 23 Uppsala, Sweden; 5 Centre for Innovation, Medical Devices and Technology, St Olav’s University Hospital, 7030 Trondheim, Norway; 6 Institute of Clinical Medicine, University of Tartu, 50406 Tartu, Estonia

**Keywords:** abdominal aortic aneurysm, SNP, MMP-9, sex, women

## Abstract

**Background/Objectives:** The etiology behind sex differences in the prevalence of abdominal aortic aneurysm (AAA) can only partly be explained by environmental factors such as smoking. Genetic factors are also likely to be part of the explanation since family history is common. We hypothesized that genetic factors on AAA prevalence might be different between the sexes. **Methods**: This study is designed as a case–control study with 83 female AAA patients, 101 female controls, 97 male AAA patients, and 196 male controls. Single nucleotide polymorphism (SNP) analysis was performed comparing 13 different SNPs. The selection of SNPs was based on previous SNP association studies, estrogen receptors, and SNPs important to inflammation and lipid metabolism, as these processes are modulated by estrogen. **Results:** A multivariable logistic regression resulted in significant differences in SNP association with AAA development between men and women in two SNPs (rs2010963 and rs8113877). Significant differences were found between cases and controls, using univariate analysis, in four SNPs: rs8113877 among women, and in rs6511720, rs2010963 and rs4988300 among men. No SNPs were significantly different compared to controls in both men and women. SNP rs8113877 is located in the promotor of the MMP-9 gene. Levels of circulating MMP-9 were measured in a subgroup of the study participants: an association between MMP-9 and AAA was found, and the association between rs8113877 and MMP-9 was sex-dependent. **Conclusions**: Genetic variability associated with AAA differs between men and women; these differences should be accounted for in future research.

## 1. Introduction

AAA affects women and men differently [1]. Men are 3–5 times more prone to develop AAA at 65–70 years of age [2,3,4]. Women tend to develop anatomically more complex aneurysms, with more angulated, shorter, and wider proximal necks [5,6,7]. Women also suffer an increased risk of rupture [8] and increased mortality after repair [9,10,11], after both elective and emergent repair [12]. Consequently, approximately 35–38% of all aneurysm-related deaths are reported in women [1,13], despite their lower prevalence.

The main pathophysiological features in AAA formation consist of a chronic inflammatory infiltrate and MMP-mediated proteolytic degradation of the extracellular matrix (ECM), with elastin and collagen being the most essential affected components [14]. Studies in rodent [15,16] models suggest that the sex difference in AAA is partly dependent on estrogen and is mediated through anti-inflammatory activity, including a reduction in MMP-9 [17]. A study by Villard et al. [18] reported on reproductive history among women without aneurysms, small aneurysms, and large aneurysms, suggesting a protective role of endogenous estrogen levels in the development of AAA.

Lipid metabolism has been identified as an essential factor in AAA development. Elevated levels of low-density lipoproteins (LDLs), decreased levels of high-density lipoproteins (HDLs), and Lipoprotein(a) are associated with an increased risk of AAA [19,20,21,22,23]. Estrogen influences lipid metabolism through different mechanisms, ranging from direct modification of receptor activity [24] to transcytosis [25].

There is a strong hereditary component in the development of non-syndromic AAA (i.e., not caused by monogenetic disease) with a reported heritability of 70–77% [26,27]. Possible sex-specific genetic mechanisms are poorly described in the literature, however.

Single-nucleotide polymorphisms (SNPs) are genetic variations in a single nucleotide occurring at a specific position in the genome among more than one per cent of the population; about 1.4 million SNPs are identified, the number increases over time.

There have been numerous studies searching for SNPs responsible for the development and prognosis of non-syndromic AAA, and the results are well summarized in two reviews [28,29]. Some results are compelling, but there is no clear pattern of genetic variation, and the identified SNPs can only explain a fraction of the heritability of AAA. We could not find any previous studies acknowledging possible interactions between sex and SNP effects, including previous GWAS studies [30,31,32,33,34].

We hypothesized that women who develop AAA may receive less protection from sex hormones, due to differences in their hormone receptors, and that such genetic differences could explain why some women do develop AAA disease. We also wanted to investigate if SNPs in pathways regulated by estrogen, which were previously associated with AAA in men, had the same effect in women. Thus, the aim was to investigate genetic variability in men and women with and without AAA.

## 2. Materials and Methods

The study was designed as a case–control study. Participants were recruited through population screening programs [2,35], due to the low prevalence of AAA among women; female cases were predominantly identified through electronic medical records and invited. All participants had an ultrasound measurement of their aorta, blood samples were collected, and the participants then completed a questionnaire on medical and family history, smoking, and current medication. In all, 477 participants were included: 83 female AAA patients, 101 female controls with a normal aorta, 97 male AAA patients, and 196 male controls. Background information is presented in Table 1.

AAA was defined as an infrarenal aortic anterior–posterior diameter ≥30 mm in women. In men, ≥35 mm was used as a threshold for AAA disease to ensure only true aneurysms were included [36]. The aim of using different definitions of AAA for men and women is to address the size discrepancy in normal aortas. The main concern was to include only true aneurysms as cases, rather than to adjust for differences in disease progression between cases.

Aortic diameters were measured by ultrasound utilizing the leading-edge-to-leading-edge principle [37].

Whole blood was kept in a −70 °C freezer where they were stored until analysis. DNA was purified from the 477 samples, and cDNA was amplified using PCR. SNP analysis was performed using a multiplate technique by LGC Genomics, KBiosciences UK Ltd., Guildford, UK. We selected 13 SNPs associated with estrogen receptors, extracellular matrix metabolism, and lipid metabolism. The SNPs were selected from previous AAA-association studies. Since no estrogen receptor SNPs were previously associated with AAA, we also included SNPs that previously have been associated with receptor-negative breast cancer, polycystic ovary syndrome (PCOS), pre-eclampsia and estrogen-associated obesity. SNPs, genes, and previous clinical associations are identified in Table 2.

Levels of circulating MMP-9 were analyzed by ELISA in all women and a randomly selected subset of men consisting of 83 cases with AAA and 85 controls. Analysis was performed using the Human MMP-9 Quantikine ELISA Kit (DMP900, R&D Systems, Minneapolis, MN, USA) and read at 450 nm using a Tecan plate reader (Tecan, Männedorf, Switzerland).

R software version 3.5.3 [38] was used for all statistics. Continuous variables are described as means, categorical variables as percentages, and *p*-values < 0.05 were considered significant. All SNPs were analyzed using an additive inheritance model.

Univariate analysis of differences in minor allele frequencies within each sex was performed. Multiple-testing correction was not performed, as the univariate analyses were not used to draw further conclusions about associations.

Logistic regression coefficients are described as OR with 95% confidence intervals, SNP alleles were considered continuous variables due to the number of minor alleles (0–2), smoking was considered a categorical variable (never/previous/active) and encoded with dummy variables in regressions.

A logistic regression with AAA prevalence as the dependent variable and sex, SNPs and smoking status as the independent variables and sex interactions between SNPs and sex and smoking status and sex were added to evaluate the differences in effects depending on sex. A second logistic regression was performed with a reduced model to reduce the risk of multicollinearity, including only SNPs with the *p*-value of either the main effect or the interaction effect below 0.1. Sex was included as an interaction term since the aim of the investigation was to characterize the sex-dependent differences in the associations between SNP and AAA development.

**Table 2:** Single-nucleotide polymorphisms (SNPs), minor alleles, genes and previous clinical associations.

Polymorphism	Minor Allele	Gene	Clinical Association	References
rs1709183	G	ESR1	Obesity	Kuźbicka [39]
rs2228480	A	ESR1	Breast cancer	Wu [40]
rs37987577	T	ESR1	Breast cancer	Sghaier [41]
rs4986938	A	ESR2	PCOS	Lidaka [42]
rs5030707	C	CST3	Aortic diameter	Maniwa [43]
rs2010963	C	VEGFA	Pre-eclampsia	Duan [44]
rs8113877	G	MMP9	AAA	Duellman [45]
rs1036095	G	TGFBR2	AAA	Baas [46]
rs764522	G	TGFBR2	AAA	Baas [46]
rs6511720	T	LDLR	AAA	Bradley [32]
rs1466535	A	LRP1	AAA	Bown [31]
rs3781590	T	LRP5	AAA	Galora [21]
rs4988300	T	LRP5	AAA	Galora [21]

## 3. Results

AAA cases and controls among both men and women differed in several known risk factors, as shown in Table 1. In univariate analyses, four SNPs were associated with AAA: rs8113877 in the MMP9 gene (39% vs. 50%, *p* = 0.04) among women, rs2010963 in the VEGFA gene (25% vs. 33%, *p* = 0.04), rs6511720 (LDLR) (6.2% vs. 12%, *p* = 0.01) and rs4988300 (LRP5) (38% vs. 50%, *p* = 0.003) among men, as shown in Table 3.

**Figure 1:**
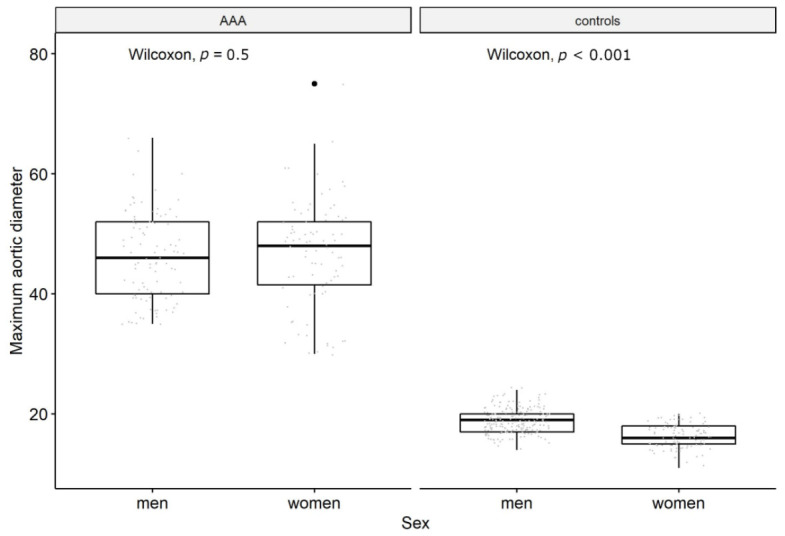
Variance in aortic diameter between groups.

Maximum AAA diameter during follow-up in women and men are plotted in Figure 1. As expected, there is a significant difference in diameter when comparing cases and controls. Among AAA cases, the range of diameters was greater among women than among the men, most likely a result of population-based screening among men [47], leading to the detection of AAA at smaller diameters and commonly operated on when reaching 55 mm.

A multivariable logistic regression with a complete set of SNPs and covariates, including interactions with sex, was performed. The results are available in Table S1. SNPs with main or interaction coefficients with a *p*-value < 0.1 were included in a reduced model.

**Figure 2:**
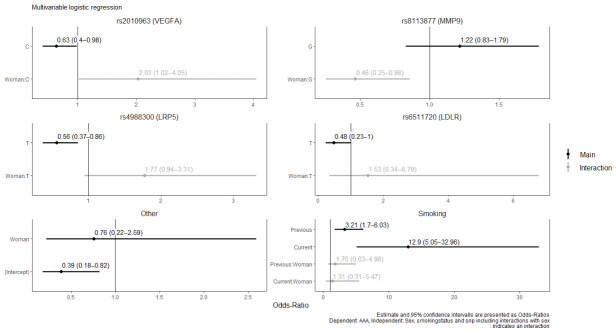
Estimates from multivariable logistic regression.

Multivariable logistic regression with the reduced model identified two SNPs rs8113877 (OR 0.46, 95% CI: 0.25–0.86, *p* = 0.01) and rs2010963 (OR 2.03, 95% CI: 1.02–4.05, *p* = 0.04) with a significant interaction between sex and allele count. Main effects, indicating the effect in men, were significant in two SNPs rs4988300 (OR 0.56, 95% CI: 0.37–0.86, *p* = 0.007), rs2010963 (OR 0.63, 95% CI: 0.4–0.98, *p* = 0.04), as shown in Figure 2.

Following the hypothesis of this investigation, further analysis was focused on the only SNP associated explicitly with AAA in women, rs8113877. It is located in the promotor region of MMP-9 and has previously been associated with AAA [45], in a 74% male cohort. Promotor activity is reported to be reduced [48] by the rs8113877 T allele, which was the major allele in the present population. To further investigate the effects, we decided to also measure MMP-9 levels in circulating plasma in all patients and controls.

MMP-9 levels were measured in a subset of the primary cohort. In agreement with previous reports, the levels of MMP-9 correlated to AAA using a univariate logistic regression (OR 1.49, 95% CI: 1.2–1.9, *p* = 0.002); when including smoking status and sex in a multivariable regression model MMP-9 presented a weaker non-significant association with AAA (OR 1.09, 95% CI: 0.71–1.67, *p* = 0.69), as shown in Table 4.

The levels of MMP-9 were not associated with rs8113877, neither in a univariate (0.03, 95% CI: −0.08–0.15, *p* = 0.56) nor in a multivariable linear regression model, including sex and smoking status (0.03, 95% CI: −0.85–0.14, *p* = 0.61). When adding an interaction between sex and rs8113877, the interaction was significant (Table 5).

There were associations with both previous smoking and current smoking. However, an *R^2^* Statistic of 0.07 indicates that other factors account for most of the variation in MMP-9 levels.

## 4. Discussion

In the current study, four SNPs were associated with AAA using univariate analysis: rs2010963 (VEGFA) rs6511720 (LDLR), rs4988300 (LRP5) among men and rs8113877 (MMP9) among women. None of the SNPs in estrogen receptor genes ESR1 or ESR2 were associated with AAA.

Multivariable logistic regression including smoking status and interaction terms with sex identified two SNPs, rs8113877 (MMP9) and rs2010963 (VEGFA), with significant interactions between sex and SNP effect, indicating that the associations between these two SNPs and AAA are modified by sex.

Main effects mirror the findings of the univariate analysis in the male group with significant effects for rs2010963 (VEGFA), rs6511720 (LDLR) and rs4988300 (LRP5).

SNP rs2010963 in the VEGFA gene has previously been associated with AAA in a Polish predominantly male cohort [48]. VEGFA is an important factor in the attraction and infiltration of leukocytes during inflammation [49], in addition to well-described effects on angiogenesis. 17-β-oestradiol in adipose tissue in part regulates VEGFA expression through estrogen receptor 1 (ESR1) [50]. Peri-aortic adipose tissue inflammation and necrosis are described as a factor in AAA development [51].

SNP rs4988300 of the LRP5 gene was associated with AAA in an Italian study by Galora et al. [21]. The level of Lipoprotein(a) was also elevated among carriers. LRP5 encodes a low-density lipoprotein receptor active in receptor-driven endocytosis. Lipoprotein(a) was suggested as a biomarker for AAA in a meta-analysis performed in 2017 [52].

SNP rs6511720 in the LDLR gene has previously been identified in a GWAS with a mixed-sex cohort (11% female cases and 49% female controls) [31]. The LDLR gene produces the simplest receptor in the LDL-receptor family. The role of LDL metabolism in AAA is not clear. However, a sizeable Mendelian randomization study from 2018 [23] supports the importance of LDL in AAA development.

SNP rs8113877 is located in the promoter region of the MMP-9 gene. Our analysis of MMP-9 levels in peripheral blood identified an association between MMP-9 levels and AAA, and that the association between rs8113877 and MMP-9 was sex-dependent.

Limitations of the study design are that we did not measure MMP-9 levels at baseline, only when AAA disease had already developed. Furthermore, we were unable to measure MMP-9 in the aortic tissue, only in the blood.

MMP-9 levels in aortic tissue are associated with AAA and are thought to be an important mechanism of ECM degradation during AAA formation [53]. The rs8113877 G allele has been identified as a genetic variant associated with lower promoter activity of the MMP-9 gene [44] in vitro. The same study by Duellman found a negative association between re811387 and AAA in a cohort of 74% males. We found that the association between MMP-9 levels and the rs8113877 polymorphism was sex-dependent. While the results should be carefully interpreted due to the limited cohort, the finding is coherent with the sex-dependent association of rs8113877 with AAA. A sex-dependent association with MMP-9 levels may explain the rs8113877 G allele’s sex-dependent association with AAA.

Hovsepian reported that plasma levels of MMP-9 directly reflected MMP-9 levels in AAA tissue [54]. However, this conclusion was based on a small sample (*n* = 4), and can be questioned.

Possible sex differences in genetic risk factors to develop AAA have not been investigated previously, partly due to the scarcity of female cases. The study population of 83 cases is small for a genetic study but large when considering other studies of women with AAA. To offset the small sample size, we reduced the number of analyzed SNPs and added robust clinical data, including detailed data on smoking, which is considered the most important environmental risk factor. Due to the limitations listed above, the selection of estrogen receptor SNPs was severely limited with a substantial risk of missing significant polymorphisms. Without tissue samples from the aortic wall, the effects of SNPs are challenging to gauge. In an attempt to verify the role of rs8113877, we measured MMP-9 levels in peripheral blood to assess the effect in vitro. A correlation between MMP-9 levels and AAA (the difference between cases and controls) was identified, but variations in rs8113877 could not explain the variability of MMP levels.

Genome-wide association studies [30,31,32] (GWAS) have identified three genetic risk loci for AAA, and a 2017 meta-analysis [33] of previous GWAS uncovered six additional risk loci. In addition to meta-analysis, an extensive bioinformatics workup was performed based on the nine identified loci. The final step of the bioinformatics analysis was a gene product interaction network, including TNF-α and the 14 genes related to the identified risk loci, which suggested MMP-9 as a central protein in the pathogenesis of aneurysm disease.

These GWAS studies and the meta-GWAS study did not address sex differences, however. In the meta-GWAS, women make up 9% of the cases and 55% of the controls. Thus, a sex difference in genetic causes for AAA may have created confounding in all these analyses.

The main limitation of this investigation is the few women with AAA. Although we invited all living women with known AAA to two large centers, we only managed to collect blood samples from 83 women with AAA. This is explained by low prevalence, the high age at which women are affected, and their poor life expectancy. The low number made it challenging to address interactions between different genes, as well as between environmental risk factors and genes. The fact that few of the patients had undergone open surgery for AAA made it impossible to study tissue samples of the AAAs. A strength of the study is that we have robust clinical data, including data on smoking habits.

## 5. Conclusions

In conclusion, we failed to verify our primary hypothesis, that genetic polymorphism of the estrogen receptors might be involved in the pathogenesis of AAA. We were able, however, to identify other sex differences in genetic variations of importance for AAA. A different genetic background in women could partly explain the observed sex differences in disease characteristics. Considering the difference in vascular biology between men and women, a difference in effects of SNPs does not seem unreasonable. Based on the results in the current study, it seems essential to consider sex differences in future genetic studies on AAA. A larger population of women with AAA is needed to assess sex-specific genetic risk factors for AAA further, and we invite other centers to collaborate with us in future projects.

## Figures and Tables

**Table 1:** Clinical characteristics.

	Women							Men						
	Cases			Controls			*p*-Value	Cases			Controls			*p*-Value
Group size (*n*)	83			101				97			196			
Age (mean)	71			70			0.22	69			69			0.28
Diseases	*n*	%	missing	*n*	%	missing		*n*	%	missing	*n*	%	missing	
Coronary artery disease	27	35	2	7	7.3	0	<0.001	28	30	0	15	7.8	0	<0.001
Diabetes	14	18	2	11	11	0	0.09	8	8.5	0	22	11	0	0.5
Claudication	12	16	0	2	2.1	0	0.001	5	5.4	0	6	3.1	0	0.3
Hypercholesterolemia	34	45	2	31	33	0	0.04	25	28	0	48	26	0	0.8
Hypertension	57	72	1	35	36	0	<0.001	57	59	0	86	44	0	0.03
COPD	16	21	1	6	6.2	0	0.004	12	12	0	7	3.6	0	0.009
Renal failure	4	5.2	2	1	1	0	0.06	2	2.1	0	1	0.52	0	0.3
CVD	16	21	0	3	3.2	0	<0.001	19	20	0	10	5.2	0	<0.001
Medication														
Treatment-ASA	33	56	0	13	14	0	<0.001	43	45	0	43	25	0	0.001
Treatment-Statins	33	57	0	21	22	0	<0.001	39	41	0	56	33	0	0.2
Smoking status			2			5							0	
Active	28	34		9	8.9		<0.001	23	24		10	5.1		<0.001
Previous	42	51		39	39			56	58		94	48		
Never	11	13		48	48			17	18		92	47		

COPD, Chronic Obstructive Pulmonary Disease; CVD, Cerebrovascular Disease; *p*-values calculated using Fischer’s exact test; missing = number of cases with missing data.

**Table 3:** Single-nucleotide polymorphisms (SNPs), genes and frequencies among women and men, with and without abdominal aortic aneurysm (AAA). Univariate analyses.

SNP (Minor Allele)	Women			Men		
	AAA (%), *n* = 76	Controls (%), *n* = 102	*p*- Values	AAA (%), *n* = 97	Controls (%), *n* = 201	*p*- Values
rs1709183 (G)	29	28	0.9	26	26	0.9
rs2228480 (A)	17	22	0.2	18	20	0.6
rs37987577 (T)	0	1.5	0.08	1.5	2.3	0.5
rs4986938 (A)	38	31	0.2	37	35	0.7
rs5030707 (C)	17	23	0.1	18	19	0.6
rs8113877 (G)	39	50	0.04	43	39	0.3
rs1036095 (G)	22	23	0.8	22	19	0.3
rs764522 (G)	16	17	0.9	14	14	1
rs2010963 (C)	30	29	0.8	25	33	0.04
rs6511720 (T)	4.8	4	0.7	6.2	12	0.01
rs1466535 (A)	39	38	0.8	33	34	0.7
rs3781590 (T)	28	31	0.6	27	29	0.6
rs4988300 (T)	43	48	0.4	38	50	0.003

*p*-values calculated using Student’s *t*-test; *n* indicates the number of study participants.

**Table 4:** Multivariable logistic regression estimates the association between AAA and MMP-9, sex, smoking habits and the interaction between sex and MMP-9.

Variable	Odds Ratio	95% Confidence Interval	*p*-Value
MMP-9	1.09	0.71–1.67	0.7
Woman	1.97	1.15–3.41	0.02
Previous smoker	4.11	2.20–8.01	<0.001
Current smoker	9.77	4.14–24.40	<0.001
Interaction Woman: MMP-9	1.35	0.77–2.43	0.3

*n* = 291; Pseudo-*R*^2^ (McFadden) = 0.166.

**Table 5:** Logistic regression estimates the association between MMP-9 levels and smoking habits, sex, rs8113877 and the interaction between rs8113877 and sex.

Variable	Coefficient	95% Confidence Interval	*p*-Value
Previous smoker	0.34	0.09–0.59	0.007
Current smoker	0.70	0.34–1.06	<0.001
Woman	−0.03	−0.27–0.20	0.8
rs8113877(G)	0.17	−0.01–0.35	0.06
Interaction Woman: rs8113877(G)	−0.24	−0.47–−0.01	0.04

N = 291; *R*^2^ = 0.067.

## Data Availability

The authors are open to share data with other researchers. Please contact the corresponding author with such requests.

## Supplementary Materials

The following supporting information can be downloaded at: https://www.mdpi.com/article/10.3390/biomedicines14051172/s1, Table S1. Complete logistic regression model, using AAA prevalence as dependent variable.

## Abbreviations

The following abbreviations are used in this manuscript:

AAA	Abdominal aortic aneurysm
SNP	Single nucleotide polymorphism
GWAS	Genome-wide association studies
MMP-9	Matrix metalloproteinase-9
ELISA	Enzyme-linked immunosorbent assay analysis
