# Supplementary material for: Women with Abdominal Aortic Aneurysms Have a Different Pattern of Genetic Variability, Compared to Men
Source: Biomedicines. 2026 May 21;14(5):1172. doi: 10.3390/biomedicines14051172 (PMC13204876; doi:10.3390/biomedicines14051172)
Supplement: Supplementary file 1 [file biomedicines-14-01172-s001.zip › biomedicines-4259931-supplementary.pdf]

Table S1. Complete logistic regression model, using AAA prevalence as dependent variable.

Variable	Odds ratio	95% Confidence interval	*p*-value
(Intercept)	0.21	0.07 - 0.61	0.005
Woman	0.30	0.04 - 2.10	0.232
rs1036095(G)	1.38	0.63 - 2.99	0.418
rs1466535(A)	1.07	0.67 - 1.72	0.775
rs1709183(G)	1.06	0.64 - 1.78	0.816
rs2010963(C)	0.62	0.35 - 1.05	0.082
rs2228480(A)	1.19	0.02 - 41.98	0.931
rs3781590(T)	0.98	0.53 - 1.81	0.941
rs3798758(T)	0.85	0.17 - 3.41	0.828
rs4986938(A)	0.95	0.58 - 1.54	0.839
rs4988300(T)	0.53	0.29 - 0.94	0.035
rs5030707(C)	0.68	0.02 - 40.85	0.854
rs6511720(T)	0.40	0.16 - 0.93	0.044
rs764522(G)	0.68	0.28 - 1.63	0.394
rs8113877(G)	1.35	0.85 - 2.15	0.208
Previous smoker	1.97	0.94 - 4.25	0.078
Current smoker	15.20	5.35 - 46.88	<0.001
Coronary artery disease	5.50	2.61 - 12.05	<0.001
Hypertension	3.41	1.91 - 6.19	<0.001
Hypercholesterolemia	0.47	0.23 - 0.91	0.028
CVD	7.21	2.85 - 19.60	<0.001
Claudication	1.51	0.44 - 5.40	0.518
COPD	1.83	0.66 - 5.10	0.247
Diabetes	0.44	0.19 - 0.99	0.053
Renal failure	1.86	0.14 - 34.04	0.653
Interaction Woman : rs1036095(G)	0.89	0.26 - 3.03	0.856
Interaction Woman : rs1466535(A)	0.99	0.44 - 2.23	0.976
Interaction Woman : rs17091838(G)	0.75	0.30 - 1.86	0.539

Variable	Odds ratio	95% Confidence interval	*p*-value
Interaction Woman : rs2010963(C)	2.27	0.98 - 5.42	0.059
Interaction Woman : rs2228480(A)	6.39	0.04 - 2068.32	0.518
Interaction Woman : rs3781590(T)	1.35	0.47 - 3.91	0.580
Interaction Woman : rs3798758(T)	0.00*	NA – NA*	0.985
Interaction Woman : rs4986938(A)	1.84	0.81 - 4.31	0.151
Interaction Woman : rs4988300(T)	1.80	0.68 - 4.71	0.233
Interaction Woman : rs5030707(C)	0.18	0.00 - 28.22	0.548
Interaction Woman : rs6511720(T)	2.36	0.40 - 13.73	0.336
Interaction Woman : rs764522(G)	0.78	0.21 - 2.88	0.704
Interaction Woman : rs8113877(G)	0.42	0.19 - 0.91	0.031
Interaction Woman : Previous smoker	3.57	0.94 - 14.92	0.069
Interaction Woman : Current smoker	2.52	0.45 - 15.13	0.299

*N = 428; Pseudo-R2 (McFadden) = 0.404; *Due to no minor alleles among women AAA cases.*
